# Supplementary material for: A prospective study of nanopore-targeted sequencing in the diagnosis of central nervous system infections
Source: Microbiol Spectr. 2024 Jan 31;12(3):e03317-23. doi: 10.1128/spectrum.03317-23 (PMC10913467; doi:10.1128/spectrum.03317-23)
Supplement: Table S1 — Serological indicators in 50 patients with suspected CNS infections. [file spectrum.03317-23-s0001.docx]

| **Supplementary table 1** Serological indicators in 50 patients with suspected CNS infections | | | | |
| --- | --- | --- | --- | --- |
|  | Peripheral blood examination | | | |
| No. | PCT (ng/ml) | IL-6 (pg/ml) | WBC (x 10 ^9^ /L) | NEC (x 10 ^9^ /L) |
| 01 | 0.15 | 33.7 | 6.34 | 3.68 |
| 02 | 0.23 | 46.4 | 6.34 | 3.68 |
| 03 | 3.31 | 22.5 | 15.77 | 14.17 |
| 04 | 1.12 | 222.4 | 28.2 | 24.9 |
| 05 | <0.05 | 110 | 8.1 | 6.6 |
| 06 | 0.06 | 25.9 | 6 | 4.6 |
| 07 | 1.56 | 18.8 | 11.58 | 10.48 |
| 08 | <0.05 | 6.4 | 7.1 | 4.2 |
| 09 | 0.48 | 15.6 | 8.6 | 7.6 |
| 10 | 0.05 | 17.8 | 14.1 | 10.8 |
| 11 | <0.05 | 10.5 | 17.01 | 15.56 |
| 12 | 0.13 | 12.2 | 17.2 | 14.1 |
| 13 | 0.68 | 533 | 16.6 | 13.6 |
| 14 | 5.27 | 21.3 | 13.49 | 12.19 |
| 15 | 7.11 | 10.5 | 13.8 | 11.4 |
| 16 | 0.11 | 11 | 11.02 | 8.91 |
| 17 | 0.57 | 557 | 18.86 | 17.81 |
| 18 | <0.05 | 14.2 | 13.99 | 12.94 |
| 19 | 0.61 | 70.3 | 16.56 | 13.71 |
| 20 | 0.74 | 60.8 | 10.5 | 8.8 |
| 21 | 0.05 | 11.2 | 8.75 | 7.19 |
| 22 | 0.09 | 39 | 10.48 | 7.84 |
| 23 | 17.4 | 12.8 | 7.93 | 5.89 |
| 24 | <0.05 | 22 | 13.2 | 11.5 |
| 25 | 0.41 | 12.8 | 7.51 | 5.9 |
| 26 | 0.11 | 6.7 | 2.1 | 0 |
| 27 | 0.7 | 51.7 | 16.7 | 14.4 |
| 28 | 1.16 | 50.3 | 17.37 | 14.26 |
| 29 | <0.05 | 26.4 | 12 | 8.9 |
| 30 | 0.11 | 12.3 | 13.79 | 12.67 |
| 31 | 1.3 | 15.8 | 26 | 21 |
| 32 | 0.18 | 14.7 | 12.3 | 11.8 |
| 33 | <0.05 | 52.9 | 8.4 | 7.22 |
| 34 | 10.8 | 22 | 10.1 | 9.3 |
| 35 | <0.05 | 11 | 13.69 | 11.02 |
| 36 | 0.27 | 399 | 14.09 | 12.36 |
| 37 | 0.11 | 34 | 8.32 | 5.94 |
| 38 | 1.18 | 42 | 13.5 | 12.6 |
| 39 | 0.12 | 17.8 | 11.5 | 10.3 |
| 40 | 0.19 | 8.1 | 22.3 | 20.9 |
| 41 | 1.34 | 2.77 | 10 | 7.8 |
| 42 | 0.98 | 23.5 | 10 | 7.33 |
| 43 | 0.64 | 1.33 | 10.3 | 7.1 |
| 44 | 0.94 | 400 | 12 | 11.3 |
| 45 | <0.05 | 27.9 | 9.6 | 6.8 |
| 46 | <0.05 | 38.1 | 12.51 | 10.59 |
| 47 | 1.02 | 34 | 12.6 | 10.8 |
| 48 | 0.17 | 2.26 | 18 | 16.71 |
| 49 | 0.59 | 76.8 | 15.89 | 14.1 |
| 50 | 0.3 | 17.4 | 8 | 7.5 |

Note: PCT, procalcitonin; IL-6, interleukin-6; WBC, white blood cell count; NEC, neutrophil count.

| **Supplement table 2** The results of CSF testing in 50 patients with suspected CNS infections. | | | | | | | |
| --- | --- | --- | --- | --- | --- | --- | --- |
| No. | CSF culture | CSF analysis | | | | | |
|  |  | TNC (per mm^3^) | CSF-TP (g/L) | CSF-Glu (mmol/L) | CSF-Cl(mmol/L) | CSF-LDH  (U/L) | CSF LA (mmol/L) |
| 01 | － | 5 | 0.28 | 3.15 | 125.6 | 14 | 1.85 |
| 02 | － | 72 | 4.47 | 0.69 | 111.1 | 2341 | 15.46 |
| 03 | － | 713 | 2.39 | 1.86 | 115.9 | 546 | 7.63 |
| 04 | － | 242 | 1.22 | 1.43 | 121.2 | 242 | 7.17 |
| 05 | － | 35 | 0.42 | 4.14 | 124.4 | 98 | 3.22 |
| 06 | － | 46 | 4.23 | 5.86 | 114.6 | 1169 | 5.71 |
| 07 | － | 151 | 3.03 | 3.9 | 123.6 | 575 | 5.4 |
| 08 | － | 75 | 0.19 | 4.05 | 123.8 | 19 | 1.9 |
| 09 | － | 121 | 3.06 | 4.28 | 145.7 | 526 | 7.75 |
| 10 | － | 656 | 8.06 | 2 | 2.49 | 120.2 | 455 |
| 11 | － | 496 | 0.85 | 3.71 | 119.1 | 109 | 2.9 |
| 12 | － | 121 | 3.06 | 4.28 | 145.7 | 526 | 7.75 |
| 13 | － | 6 | 1.64 | 5 | 139.8 | 199 | 2.91 |
| 14 | － | 1151 | 1.64 | 1.73 | 114.6 | 113 | 6.67 |
| 15 | － | 8188 | 2.64 | 0.43 | 116.6 | 1933 | 20.1 |
| 16 | － | 2 | 0.59 | 3.46 | 128.6 | 10 | 1.81 |
| 17 | － | 29 | 1.96 | 2.13 | 136.4 | 227 | 6.6 |
| 18 | － | 268 | 5.12 | 1.39 | 0.8 | 117.1 | 256 |
| 19 | － | 10 | 1.37 | 5.94 | 137.5 | 347 | 6.46 |
| 20 | － | 479 | 3.06 | 2.86 | 119.8 | 451 | 4.25 |
| 21 | － | 90 | 1.19 | 4.41 | 123.1 | 116 | 3.22 |
| 22 | － | 7 | 0.44 | 3.83 | 122.9 | 17 | 1.8 |
| 23 | ＋ | 17 | 0.5 | 3.02 | 122.4 | 13 | 2.73 |
| 24 | － | 6 | 0.45 | 3.35 | 129.1 | 45 | 1.69 |
| 25 | － | 395 | 0.79 | 1.93 | 109.1 | 84 | 4.99 |
| 26 | － | 5 | 0.18 | 3.27 | 123.4 | 18 | 1.27 |
| 27 | － | 270 | 0.96 | 2.79 | 121.8 | 94 | 3.66 |
| 28 | ＋ | 1295 | 3.87 | 0.03 | 104.6 | 467 | 15.55 |
| 29 | ＋ | 12 | 1.68 | 0.01 | 112.6 | 18 | 1.95 |
| 30 | － | 1 | 0.3 | 6.99 | 119.5 | 9 | 1.94 |
| 31 | ＋ | 25 | 1.75 | 3.62 | 127.7 | 82 | 2.5 |
| 32 | ＋ | 80 | 0.25 | 3.09 | 128.9 | 30 | 2.13 |
| 33 | － | 56 | 5.05 | 2 | 1.82 | 119.3 | 3.54 |
| 34 | － | 1477 | 1.02 | 0.66 | 115.8 | 250 | 7.35 |
| 35 | － | 590 | 0.77 | 2.21 | 116.7 | 96 | 5.79 |
| 36 | － | 9 | 0.5 | 5.47 | 128.1 | 38 | 3.08 |
| 37 | － | 41 | 1.19 | 2.46 | 117.4 | 46 | 3.53 |
| 38 | － | 1021 | 0.56 | 3.24 | 119.1 | 75 | 6.6 |
| 39 | ＋ | 12649 | 3.05 | 0.04 | 101.5 | 1704 | 12.82 |
| 40 | ＋ | 1511 | 17.92 | <1 | 2.4 | 117.7 | 678 |
| 41 | － | 10234 | 9.17 | 2 | 2.05 | 126.6 | 268 |
| 42 | － | 8436 | 2 | 0.85 | 116 | 1303 | 14.35 |
| 43 | － | 395 | 1.14 | 1.22 | 123.6 | 280 | 5.24 |
| 44 | － | 741 | 1.86 | 4.17 | 143.6 | 212 | 6.05 |
| 45 | － | 1026 | 0.87 | 2.02 | 122.6 | 36 | 4.78 |
| 46 | － | 3 | 0.19 | 4.31 | 118.9 | 11 | 3.42 |
| 47 | － | 1177 | 2.43 | 0.88 | 116.2 | 157 | 14.93 |
| 48 | ＋ | 1620 | 3 | 0.92 | 126 | 329 | 15.33 |
| 49 | － | 5627 | 2.74 | 1.14 | 123.3 | 1051 | 10.66 |
| 50 | － | 31 | 0.7 | 2.06 | 121.6 | 35 | 2.97 |

Note: TNC, total nucleated cell; CSF-TP, total protein in cerebrospinal fluid; CSF-Glu, glucose in cerebrospinal fluid; CSF-Cl, chloride in cerebrospinal fluid; CSF-LDH, lactate dehydrogenase in cerebrospinal fluid; CSF-LA, lactate levels in cerebrospinal fluid.
